# Extensive genome analysis identifies novel plasmid families in *Clostridium perfringens*

**DOI:** 10.1099/mgen.0.000995

**Published:** 2023-04-20

**Authors:** Emily L. Gulliver, Vicki Adams, Vanessa Rossetto Marcelino, Jodee Gould, Emily L. Rutten, David R. Powell, Remy B. Young, Gemma L. D’Adamo, Jamia Hemphill, Sean M. Solari, Sarah A. Revitt-Mills, Samantha Munn, Thanavit Jirapanjawat, Chris Greening, Jennifer C. Boer, Katie L. Flanagan, Magne Kaldhusdal, Magdalena Plebanski, Katherine B. Gibney, Robert J. Moore, Julian I. Rood, Samuel C. Forster

**Affiliations:** ^1^​ Centre of Innate Immunity and Infectious Disease, Hudson Institute of Medical Research, Clayton, VIC, 3168, Australia; ^2^​ Department of Molecular and Translational Sciences, Monash University, Clayton, VIC, 3800, Australia; ^3^​ Department of Microbiology, Infection Program, Biomedicine Discovery Institute, Monash University, Clayton, VIC, 3800, Australia; ^4^​ Monash Bioinformatics Platform, Monash University, Clayton, VIC, 3800, Australia; ^5^​ Centre to Impact AMR, Monash University, Clayton, VIC, 3800, Australia; ^6^​ School of Health and Biomedical Sciences, RMIT University, Bundoora, VIC, 3083, Australia; ^7^​ Tasmanian Vaccine Trial Centre, Clifford Craig Foundation, Launceston General Hospital, Launceston, TAS, 7250, Australia; ^8^​ School of Medicine, Faculty of Health Sciences, University of Tasmania, Launceston, TAS, Australia; ^9^​ Department of Food Safety and Animal Health, Norwegian Veterinary Institute, PO Box 750, Sentrum, 0106, Oslo, Norway; ^10^​ Department of Infectious Diseases, University of Melbourne, at the Peter Doherty Institute for Infection and Immunity, Melbourne, VIC, Australia; ^11^​ School of Science, RMIT University, Bundoora West Campus, Bundoora, VIC, Australia

**Keywords:** *Clostridium perfringens*, genome, plasmids, toxins, horizontal gene transfer, phylogeny

## Abstract

Globally, the anaerobic bacterium *Clostridium perfringens* causes severe disease in a wide array of hosts; however, *C. perfringens* strains are also carried asymptomatically. Accessory genes are responsible for much of the observed phenotypic variation and virulence within this species, with toxins frequently encoded on conjugative plasmids and many isolates carrying up to 10 plasmids. Despite this unusual biology, current genomic analyses have largely excluded isolates from healthy hosts or environmental sources. Accessory genomes, including plasmids, also have often been excluded from broader scale phylogenetic investigations. Here we interrogate a comprehensive collection of 464 *C *. *perfringens* genomes and identify the first putative non-conjugative enterotoxin (CPE)-encoding plasmids and a putative novel conjugative locus (Bcp) with sequence similarity to a locus reported from *Clostridium botulinum *. We sequenced and archived 102 new *C. perfringens* genomes, including those from rarely sequenced toxinotype B, C, D and E isolates. Long-read sequencing of 11 *C *. *perfringens* strains representing all toxinotypes (A–G) identified 55 plasmids from nine distinct plasmid groups. Interrogation of the 464 genomes in this collection identified 1045 plasmid-like contigs from the nine plasmid families, with a wide distribution across the *C. perfringens* isolates. Plasmids and plasmid diversity play an essential role in *C. perfringens* pathogenicity and broader biology. We have expanded the *C. perfringens* genome collection to include temporal, spatial and phenotypically diverse isolates including those carried asymptomatically in the gastrointestinal microbiome. This analysis has resulted in the identification of novel *C. perfringens* plasmids whilst providing a comprehensive understanding of species diversity.

## Data Summary

The genomes used in this study are publicly available (Table S2, available in the online version of this article) and can be retrieved from the European Nucleotide Archive (ENA; https://www.ebi.ac.uk/ena/browser/) under study number PRJEB55793. All supporting data are available in supporting tables.

**Impact Statement**

*
Clostridium perfringens* is a well-known human and animal pathogen, causing food poisoning, enteric disease and myonecrosis across diverse host species. Strains are also commonly found asymptomatically within the healthy human gastrointestinal microbiome, colonizing in the first year after birth and are found throughout life. Isolates of this species can carry up to 10 plasmids, making it an important model for studying plasmid biology. In this work, we assemble the largest collection of *C. perfringens* genomes (*n*=464) to investigate the genomic diversity and identify novel plasmids within this important species. This collection includes the first closed genome sequenced isolates from toxinotype B and D strains, in addition to closed genomes from each of the toxinotypes (A–G). This work demonstrates that human commensal strains are not phylogenetically distinct from pathogenic strains, other than by plasmid carriage. Extensive plasmid analysis of isolates in this collection has identified key relationships between host species and *C. perfringens* strains and identified a putative novel conjugation locus (Bcp) with sequence similarity to *Clostridium botulinum* plasmids.

## Introduction

*Clostridium perfringens* is a Gram-positive anaerobic bacterium that demonstrates a capacity to infect multiple body sites in both humans and domestic livestock. It can cause conditions ranging from food poisoning [1], enteric disease [2] and myonecrosis [3], across a vast host range [4–6]. However, *C. perfringens* exhibits an extensive environmental distribution and also exists as a component of the normal health-associated gastrointestinal microbiome [7, 8]. Isolates are classified by toxinotypes (A–G) based on the presence of combinations of six toxins: alpha toxin, beta toxin, epsilon toxin, iota toxin, *Clostridium perfringens* enterotoxin (CPE) and NetB [9]. These toxins, and additional toxins and extracellular enzymes (henceforth referred to as toxins for simplicity), may determine the host-specific disease manifestations [2, 10]. For example, the combination of *cpb* and *etx* in toxinotype B strains is correlated with disease in sheep [11] and the presence of *netB* in toxinotype G strains is associated with disease in chickens [5].

Plasmids are critical for *C. perfringens* disease pathogenesis, with the toxinotypes largely determined by plasmid-encoded toxins. Additionally, individual isolates may carry many different or closely related plasmids [12]. Currently, there are three characterized plasmid groups, ranging from small (10 kb) non-conjugative pIP404 family members [13], to the large (>100 kb) Tcp and Pcp conjugative plasmid families [13, 14]. The well-characterized tetracycline resistance plasmid pCW3, and many closely related toxin plasmids, are included in the Tcp group [15–18]. The Pcp group includes the conjugative plasmid pCP13 [19], which carries the *cpb2,* beta2 toxin gene, and two other Pcp group plasmids that encode a novel binary enterotoxin, BecAB [20].

Targeted genome-wide phylogenetic analysis provides the capacity to understand these host-specific disease relationships, yet existing analysis has largely focused on specific pathogenic strains from one disease host or phenotype [21–25] or has only just recently begun to include rarely sequenced toxinotypes, such as B, C, D and E strains [26–28]. Large-scale genomic studies of *C. perfringens* have suggested that there are either four [27] or five [26] clades, two of which are disease-specific, whilst the remaining three are independent of the host or disease caused by isolates. While species-wide genomic plasmid analysis has not been undertaken, horizontally transferred introns and integrases have been identified [26]. Given the important role plasmids play in *C. perfringens* biology, due to their carriage of disease-causing toxins, and the largely uncharacterized interplay between phylogenetic diversity and plasmid families, further genomic investigation is essential. The present study uses a diverse collection of 464 *C *. *perfringens* genomes to determine phylogeny and plasmid diversity. This analysis has enabled the identification of novel plasmid groups, including a novel conjugation locus.

## Methods

### Bacterial culturing, DNA preparation and sequencing

A total of 102 previously unsequenced *C. perfringens* strains were collected from a variety of sources (Table S2). Strains tested were cultured on YCFA agar [29] at 37 °C, overnight, in anaerobic conditions and subcultured to check for purity, before being grown in YCFA broth overnight at 37 °C. Cells were pelleted by centrifugation at 4000 ***g*** for 10 min and washed with PBS solution prior to DNA extraction. For short-read sequencing, DNA was extracted from 20 ml bacterial pellets using the FastPrep spin kit for soil DNA extraction (MPBio), following the manufacturer’s instructions. Genomic DNA libraries were prepared using the Illumina Nextera XT DNA library prep kit and sequenced using the Illumina NextSeq550 at the MHTP medical genomics facility (Table S2). Other samples were extracted using either a CsCl gradient followed by an isopropanol extraction as per Abraham and Rood [30] or by alkaline lysis, followed by a chloroform extraction and ethanol precipitation as per O'Connor *et al*. [31]. These samples were then sequenced on an Illumina HiSeq 4000 at the Wellcome Sanger Institute (Table S2). The remaining samples were extracted using alkaline lysis and sequenced on the Illumina MiSeq at Micromon Genomics (Table S2).

For long-read sequencing, bacteria were grown in 10 ml of YCFA broth culture overnight, at 37 °C in anaerobic conditions. DNA was extracted from 1 ml bacterial pellets that were collected by centrifugation at 4000 ***g*** for 5 min, using the MasterPure DNA extraction kit for Gram-positive bacteria (Lucigen), following the manufacturer’s instructions. Libraries were prepared using the Nanopore ligation sequencing kit and sequenced on the Oxford Nanopore MinION. Plasmid DNA from *C. perfringens* strain CN4003 was extracted using a Large-Construct kit (Qiagen) and sequenced at the University of Melbourne using PacBIO technology.

### Genome collection, assembly and annotation

To generate a larger collection of genomes for analysis beyond the 102 previously unsequenced isolates, the ENA database was used to search for any publicly available *C. perfringens* genomes from before December 2021. For short-read sequencing, the resultant sequencing files and those collected from ENA were trimmed using Trimmomatic v0.38 using the default parameters and assembled using SPAdes v3.13.0. For long-read sequencing, the long and short reads from each strain were assembled using the hybrid assembly approach and default parameters in Unicycler v0.4.8 [32].

The genomes were then checked for quality using CheckM v1.1.3 [33] and assembly-stats 1.0.1 [34], where genomes were considered of high quality if they showed >90 % completeness with <5 % contamination. The genome taxonomy was also examined using CheckM and GTDB-Tk identify, align and classify using default parameters [35], where only genomes identified as *C. perfringens* were included. A total of 464 high-quality genomes were identified (Table S1) and annotated using Bakta v1.5.1 [36] to be used for phylogenetic analyses. Gene ontology was determined using Biocyc [37]. All sequencing and assembly files were uploaded to the ENA repository (PRJEB55793).

### Phylogenetic and distance characterization

To determine the phylogenetic relationships between strains, genome annotations were compared and aligned using the MAFFT alignment software within Roary v3.13.0 [38]. Core genes were designated as genes found in 99 % of genomes. The alignment of core genes then was used to reconstruct a phylogenetic tree using RAxML v8.2.11 (-m GTRGAMMA -p 12345) [39]. For whole genome characterization, genomic distance trees were reconstructed using MASHtree v1.2.0 [40]. The phylogenetic and distance trees were visualiszed using the interactive Tree of Life (iTOL) [41]. Clades within trees were determined as strains within a clade which had 97.5 % sequence similarity as determined by dRep v3.4.1. Functional analysis of core genes was performed using BioCyc [37]. The core gene list was determined to have enriched gene ontology terms using a Fisher exact test with Benjamini–Hochberg correction where enrichment was seen at *q*<0.05.

A BLASTp v2.9.0+ [42] search was used to identify the presence of specific protein-coding sequences in genomes, such as toxin, MGE and bacitracin resistance and Rep proteins (Table S1), whereas antimicrobial resistance genes and the presence of *pfoA* and *alv* were identified using ABRicate v0.7 [43] with the resfinder database and custom *C. perfringens* genome databases respectively, using default parameters [44]. This information and other metadata such as the source and toxinotype of each strain were mapped to the trees using iTOL [41]. All statistical calculations of gene or clade enrichment were performed using a Fisher exact test with a cut-off of *P*<0.05.

### Plasmid analysis

Annotated genomes used for plasmid analysis were those assembled with both long and short reads (Table 1). Plasmids were identified as closed contigs encoding a *rep* gene. Comparisons between plasmids were made using EasyFig. v2.2.3[45] and BRIG v0.95 [46]. Genes of interest were aligned using MAFFT v7.453 [47] and distance trees were reconstructed using FastTree v2.1.11 [48]. Plasmid contigs were identified through an iterative Blastp v2.9.0+ [49] search of all genomes encoding Rep proteins. The identified plasmid contigs were extracted and amino acid sequences were examined using Blastp v2.9.0+ [49] for toxin, antimicrobial resistance and bacteriocin protein-coding sequences. Heatmaps were generated using the pheatmap v1.0.12 R package, without clustering or standardizing. Plasmids were determined to be novel if they showed <30 % sequence similarity to any plasmids in known plasmid groups.

**Table 1.** Closed plasmids generated and analysed in this study

Strain	Toxinotype	Plasmid name	Size (bp)	Toxin genes	Antimicrobial resistance (gene)	Bacteriocin gene	Plasmid group (subgroup)
CP24_03	A	pCP24_03_2	71520				Tcp
		pCP24_03_3	67289				Pcp
		pCP24_03_4	12729				pIP404
		pCP24_03_5	4 438				Small (2)
		pCP24_03_6	4 427				Small (2)
		pCP24_03_7	4 159				Small (1)
JIR12688	A	pJIR12688_2	12 133			*uviAB*	pIP404
		pJIR12688_3	11802			*hlyD*	pIP404
		pJIR12688_4	4 157				Small (1)
TCP018	A	pTCP018_3	57984				Pcp
		pTCP018_9	4 383				Small (3)
		pTCP018_10	3 842				Small (3)
		pTCP018_11	3200				Small (3)
JGS1984	B	pJGS1984_2	94569	*cpb, tpeL*			Tcp
		pJGS1984_3	72689				Pcp
		pJGS1984_4	65688	*etx, cpb2atyp*†		*albA*	Tcp
		pJGS1984_5	45105				Bcp
		pJGS1984_6	11 889			*hlyD*	pIP404
		pJGS1984_7	10529			*hlyD*	pIP404
		pJGS1984_8	4 054				Small (4)
CN5383	C	pCN5383_6	120789	*cpb*		*uviAB*	Tcp
		pCN5383_7	53913	*tpeL*			Tcp
		pCN5383_8	46844				Phage-like
CN4003	D	pJIR4163	82149	*cpb2*			Tcp
		pJIR4165	100031	cpe becAB*			Tcp
		pJIR4714	66598				Pcp
		pJIR4164	73803	*etx*			Tcp
		pJIR4168	81681	*lam*			Tcp
		pJIR4167	5 254				Small (3)
		pJIR4166	2 671				Small (2)
JGS4138	D	pJGS4138_5	101610	cpe, becAB*		*albA*	Tcp
		pJGS4138_6	97996	*etx*		*albA*	Tcp
		pJGS4138_7	91259				Pcp
		pJGS4138_8	82090	*cpb2atyp*†			Tcp
58 875	E	p58875_2	102972				Pcp
		p58875_3	71255				Tcp
		p58875_4	61893				Pcp
		p58875_5	57901	*cpb2atyp*†			Tcp
		p58875_6	57472	iap/iab, cpe*			Tcp
		p58875_7	46991				Tcp
		p58875_8	46324				Bcp
ATCC 27324	E	pATCC27324_2	110299	iap/iab, cpe*		*hlyD*	Tcp
		pATCC27324_3	83200	*cpb2atyp*†		*bcn*	pIP404
		pATCC27324_4	63402				Pcp
		pATCC27324_5	53129				Tcp
		pATCC27324_6	3 639				Small (2)
		pATCC27324_7	3 397				Small (2)
JIR13122	F	pJIR13122_3	72713	*netE netF*			Tcp
		pJIR13122_4	48590	*cpe cpb2*			Tcp
		pJIR13122_5	47234		Tetracycline [*tetA*(P), *tetB*(P)]		Tcp
		pJIR13122_6	4 410				Small (3)
NE18	G	pJIR3536	82128	*netB*			Tcp
		pJIR3844	70183	*cpb2atyp*†			Tcp
		pJIR3527	48781		Tetracycline [*tetA*(P), *tetB*(P)]		Tcp
		pJIR3843	3 201				Small (3)

*Toxin genes are fragmented.

†Atypical *cpb2* gene.

## Results

### Expansion of the *C. perfringens* genome collection confirms five phylogenetic clades

**Fig. 1.**
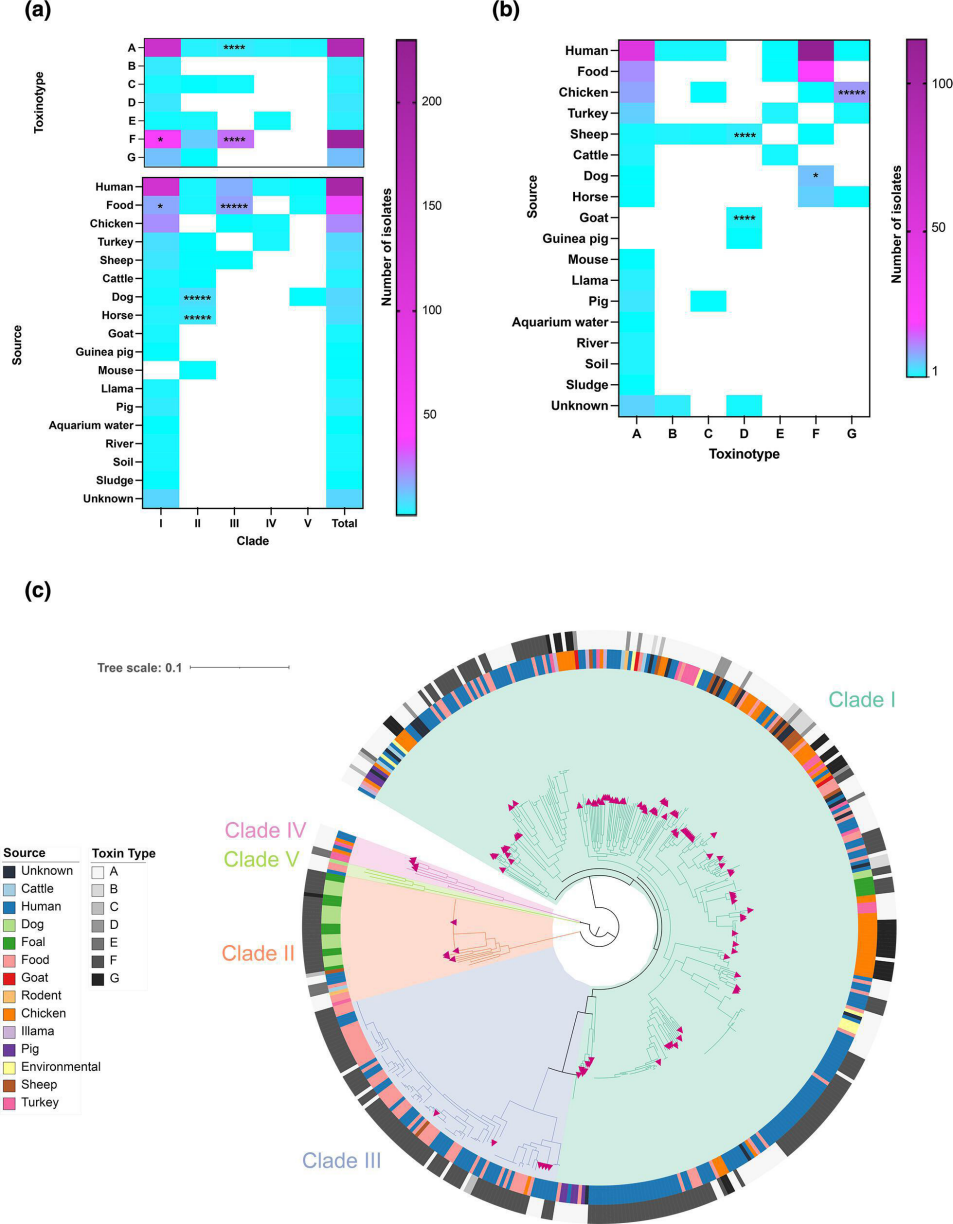
Distribution of *C. perfringens* isolates across species, clades, toxinotypes and phylogeny, where scale is branch length which is defined as base pair substitutions per sequence site. Heatmaps showing (**a**) the number of isolates in each clade per source and toxinotype and (**b**) the number of isolates from each source per toxinotype. Significantly overrepresented groups are indicated, as determined by Fisher’s exact test, *P<0.05, **P<0.01, ***P<0.001, ****P<0.0001, *****P<0.00001. (**c**) Core gene phylogeny of 464 *C *. *perfringens* strains with toxinotype (outer ring), source (inner ring) and strains sequenced in this study (pink triangles).

To determine the genomic diversity of *C. perfringens,* the genomes of 102 previously unsequenced strains isolated from environmental locations, food sources and 10 different host species (humans, chickens, turkeys, sheep, cattle, dogs, goats, guinea pigs, llamas and pigs) were sequenced (Table S2). These strains were representative of all toxinotypes (70 type A, 10 type B, two type C, 11 type D, three type E, three type F and three type G strains) collected from six continents over seven decades (Fig. 1a, b, Table S2).

**Fig. 2.**
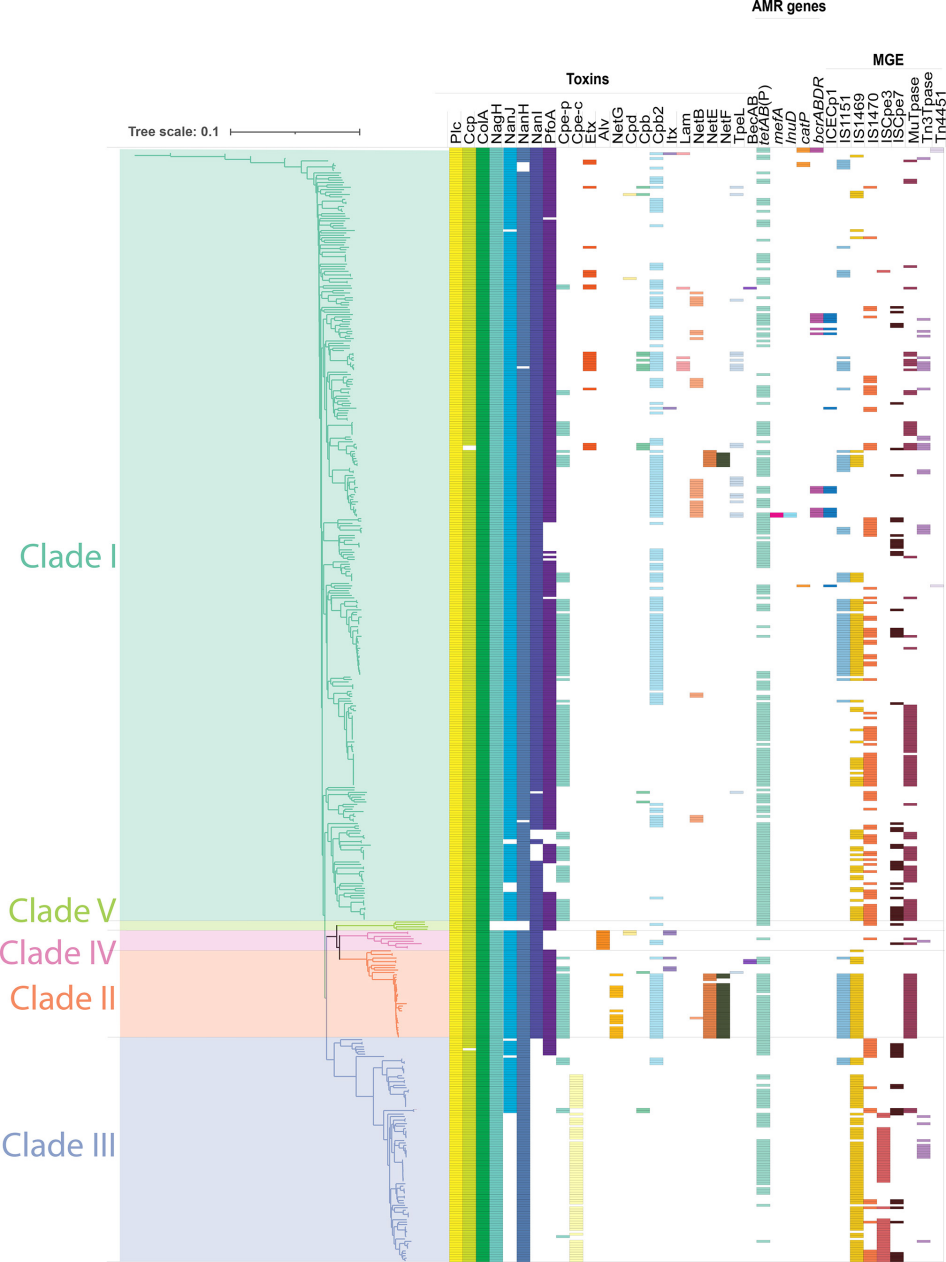
Toxin, AMR and MGE genes are widespread throughout the *C. perfringens* phylogeny. Genome distance tree generated from the whole genome sequences of 464 *C. perfringens* isolates, where scale is branch length which is defined as base pair substitutions per sequence site. Each strain is annotated with clade (branch colours and highlights), and their toxin, antimicrobial resistance (AMR) genes and mobile genetic elements (MGEs) (boxes right of the tree).

Combining our collection with publicly available genomes resulted in an analysis set of 464 genomes with a pangenome of 28 042 genes, of which 1311 were core genes (i.e. present in 99 % of isolates, Table S3). The core genes were statistically enriched for 34 functions (*q*<0.05; Fisher’s exact test, Benjamini–Hochberg correction), including metabolism, RNA processing and DNA repair (Table S4). Additionally, the virulence-related gene *argD* was identified as a core gene (*n*=464, 100 %), whilst the cognate endopeptidase-encoding gene *argB* was only identified in 89 % of isolates (414/464). Furthermore, the *virR/virS* regulatory genes were also widely distributed in these isolates (*n*=449, 96 % and *n*=398, 85 % respectively), but were not a part of the core *C. perfringens* genome. Both core gene and whole-genome phylogenies identified five distinct clades of *C. perfringens* (average nucleotide identity 97.5 %) with identical strain locations within clades (Figs 1c and 2, Table S2). Clade I was the most extensively sequenced clade, with 330 strains, from 17 sources representing all toxinotypes. Clades II, III, IV and V were represented by 37, 85, eight and four strains, respectively. All clades contained strains isolated from humans, and they also all contained isolates of toxinotype A. In addition, clades I and III were enriched for strains isolated from food (Fisher’s exact test *P*=0.02 and *P*<0.00001, respectively) and of toxinotype F (Fisher’s exact test *P*=0.03 and *P*=0.0004, respectively), as expected for human food poisoning isolates, although clade III was also enriched for strains of toxinotype A (Fisher’s exact test *P=*0.0001). Clade II was enriched for strains isolated from dogs (Fisher’s exact test *P*<0.00001) and foals (Fisher’s exact test *P*<0.00001; Fig. 1a, c), although 25 of the 27 dog and foal strains were isolated in the same study.

Reported correlations between *C. perfringens* toxins and disease hosts were also seen in this dataset [2, 5, 9, 50]; whilst some toxinotype groups only contained a small number isolates, the statistical analysis identified correlations between strains of toxinotype B and sheep hosts (Fisher’s exact test *P*=0.0067) [11, 51, 52], type D strains and goat and sheep hosts (Fisher’s exact test *P*<0.0004 and *P*<0.0001, respectively) [4, 53], type F strains and dogs (Fisher’s exact test *P*<0.0301) [54, 55], and type G strains and chickens (Fisher’s exact test *P*<0.00001) [9] (Fig. 1b). Furthermore, as previously noted, there was no association between type E strains and a disease phenotype [2].

### Whole genome analysis identifies horizontal gene transfer driving phenotypic differentiation

To understand the relationship between *C. perfringens* phylogeny and accessory genes, we first focused on toxin genes. To date, *C. perfringens* strains have been shown to produce more than 21 different toxins or extracellular enzymes, many of which are plasmid-encoded [56]. Hence, all of the toxin genes used for typing, with the exception of the alpha toxin gene (*plc*), can be plasmid-encoded. Analysis of the amino acid sequences encoded by these 21 toxin genes showed that the chromosomally encoded *plc* (also known as *cpa*) and *colA* genes were present in every isolate (*n*=464; 100 %) and the chromosomally encoded *ccp* (*n*=461; 99.4 %), *nagH* (*n*=460; 99.1 %)*, nanJ* (*n*=389; 83.8 %)*, nanH* (*n*=458; 98.7 %), *nanI* (*n*=359; 77.4 %) and *pfoA* (*n*=336; 72.4 %) were also highly represented. Given the genes for *nanJ, nanH* and *nanI* are encoded on the same operon along with the *nanR* regulator (*n*=363, 78.2 %), these results indicate that in some isolates they only encode parts of this operon. The most prevalent of the plasmid-encoded genes was *cpb2* (*n*=183; 39.4 %), while the only toxin gene found encoded both chromosomally and on plasmids, *cpe*, was identified in 46.8 % of isolates (*n*=217) (Fig. 2).

To understand the relationship between the genomic location of these toxin genes and their clade distribution, their phylogenetic location was determined (Fig. 2). Chromosomally encoded toxins were found in one of three states: found in all strains, found significantly enriched within a clade or sporadically lost in a phylogenetically independent manner. Those found in all strains were *plc* and *colA*; those found to be significantly enriched within clades were *nanI* (absent in clade III: *P<*0.00001), *pfoA* (present in all but clade IV: *P*=0.0238), *alv* (absent in clade V: *P<*0.00001) and chromosomally encoded *cpe* (only found in clade III: *P<*0.00001), and those found to be sporadically lost in a phylogenetically independent manner were *ccp, nanJ* and *nagH.* Plasmid-encoded toxins (*etx*, *cpd, becAB*, *cpb, cpb2, tpeL, itx, lam, netB, netE, netF, becAB* and plasmid-encoded *cpe*) were found without significant enrichment within clades, except for *netG* which was only found in clade II (*P<*0.00001). However, as all the *netG*-encoding strains were derived from the same study, this clade specificity may be due to sampling limitations [6].

### Antimicrobial resistance genes are distributed across *C. perfringens* clades

In addition to toxin genes, antimicrobial resistance genes were also found within the *C. perfringens* accessory genome. The ABRicate pipeline [43], coupled with a targeted blast-based search for known *C. perfringens* bacitracin resistance protein-coding sequences [57], identified genes potentially conferring resistance to tetracyclines (270 isolates), bacitracin (15 isolates), chloramphenicol (five isolates), macrolides (two isolates) and lincosamides (two isolates) (Fig. 2). Except for tetracycline resistance, the carriage of antibiotic resistance was not common amongst strains of *C. perfringens* and did not correlate with the genetic relatedness of strains. Taken together, these results indicate that horizontal gene transfer plays a substantial role in driving accessory gene composition and the disease phenotype within *C. perfringens *.

### Extensive mobile genetic elements account for large accessory genome

*C. perfringens* is known to contain numerous diverse mobile genetic elements (MGEs), including integrative conjugative elements (ICEs), integrative mobilizable elements (IMEs), insertion sequences (IS) and transposons [26, 57]. Nine key MGEs were identified in the 464 genomes, including 16 ICE*cp1* variants, 97 elements related to IS*1151*, 190 to IS*1469*, 324 to IS*1470*, 59 to IS*cpe3*, 266 to IS*cpe7* and three Tn*4451*-like elements (Fig. 2). In addition, there were 125 Mu-like transposase genes and 35 Tn*3*-like transposase genes. These elements and genes were found across all clades, with the exception of the absences of IS*1151* from clades IV or V, and Tn*3*-like transposase genes from clades II and V, as well as the presence of ICE*cp1* and Tn*4451* only in clade I (Fig. 2). The mobile elements were also found across multiple toxinotypes, with the broadest toxinotype coverage from IS*1170,* found in all isolates, followed by IS*1151* and Mu-like transposases found in six toxinotypes (all except C and all except E, respectively), IS*1469* and Tn*3*-like transposase found in five (missing from B and D strains and missing from C and E strains, respectively), IS*cpe7* found in four (toxinotypes A, B, C and F), ICE*cp1* and IS*cpe3* found in three (toxinotypes A, E and G and toxinotypes A, D and F, respectively), whilst Tn*4451* was only found in isolates from toxinotype A. This analysis confirms that putative ICEs, IMEs, IS and transposons are found broadly, throughout *C. perfringens,* irrespective of clade or toxinotype.

### Detailed characterization of plasmids from 11 isolates reveals novel *C. perfringens* plasmid groups

*C. perfringens* is known to carry many plasmids, including both small plasmids and large conjugative plasmids that can encode toxin and antimicrobial resistance genes. Sequencing of the large conjugative plasmids presents a challenge [58] as strains can carry multiple (up to seven within a single strain in the current study), closely related, large (45–140 kb in size) conjugative plasmids [12, 14]. These plasmids are usually low-copy number and can share ~35 kb of sequence [15, 58, 59], making assembly of short-read sequencing problematic. To overcome these issues, hybrid long-read and short-read sequencing of 11 strains, representing all toxinotypes (A–G), was performed (Table 1). Analysis of these data identified 55 closed plasmid sequences varying from 2671 to 120789 bp in size (Table 1), which encoded 2852 genes, of which at least 1162 (40.7 %) were hypothetical. When each plasmid was assessed for the presence of toxin and antimicrobial resistance genes, two antimicrobial resistance loci and 11 of 13 known plasmid-associated toxin genes were identified (Table 1). The presence of these toxin-plasmids correlated with the toxinotype of each strain where the type A strains carried no toxin-plasmids, the type B strain carried both a *cpb* and *tpeL* plasmid and an *etx* and atypical *cpb2* plasmid, the type C strain carried both a *cpb* plasmid and a *tpeL* plasmid, and the type D strains both carried three toxin plasmids, a *cpe* and *becAB* plasmid, an *etx* plasmid, and a *cpb2* plasmid. One of the type D strains, CN4003, also carried a fourth toxin plasmid encoding the *lam* toxin. The type E strains carried a *cpb2* plasmid and an *iap*/*ibp* (encoding iota toxin) and highly fragmented *cpe* plasmid, the type F strains carried a *cpe* and *cpb2* plasmid and a *netE* and *netF*-containing plasmid, and the type G strain carried *netB* and *cpb2* plasmids. One type A strain (CP24_03) was previously characterized as carrying a plasmid-associated delta toxin gene, *cpd* [60]; however, *cpd* was found chromosomally, flanked by an IS*110* family transposase, indicating that it may be present on a chromosomally inserted, transposable element.

The *cpe* genes were located on three large plasmids, two of which appeared to be non-conjugative and displayed 99.7 % nucleotide sequence identity. The strains that contained these two plasmids showed 97 % whole genome identity, despite being isolated 50 years apart on different continents. Strain CN4003 was isolated in Northern Ireland from a lamb in 1956, and JGS4138 was isolated from a goat in the USA in 2002. Both strains are type D isolates and the unique region flanking the CN4003 *cpe* gene had been previously noted [61], but the plasmid was not fully sequenced at that time. These *cpe* plasmids also contained highly fragmented *becAB* genes and a *tcpM* gene, which is commonly carried by Tcp plasmids. The remainder of the Tcp locus was missing and no other conjugation-related proteins could be identified, suggesting that extensive rearrangement had occurred to generate these plasmids. These findings are the first identification of *cpe* genes located on what appears to be non-conjugative plasmids.

### Plasmid classification identifies a novel, potentially conjugative, plasmid group

**Fig. 3.**
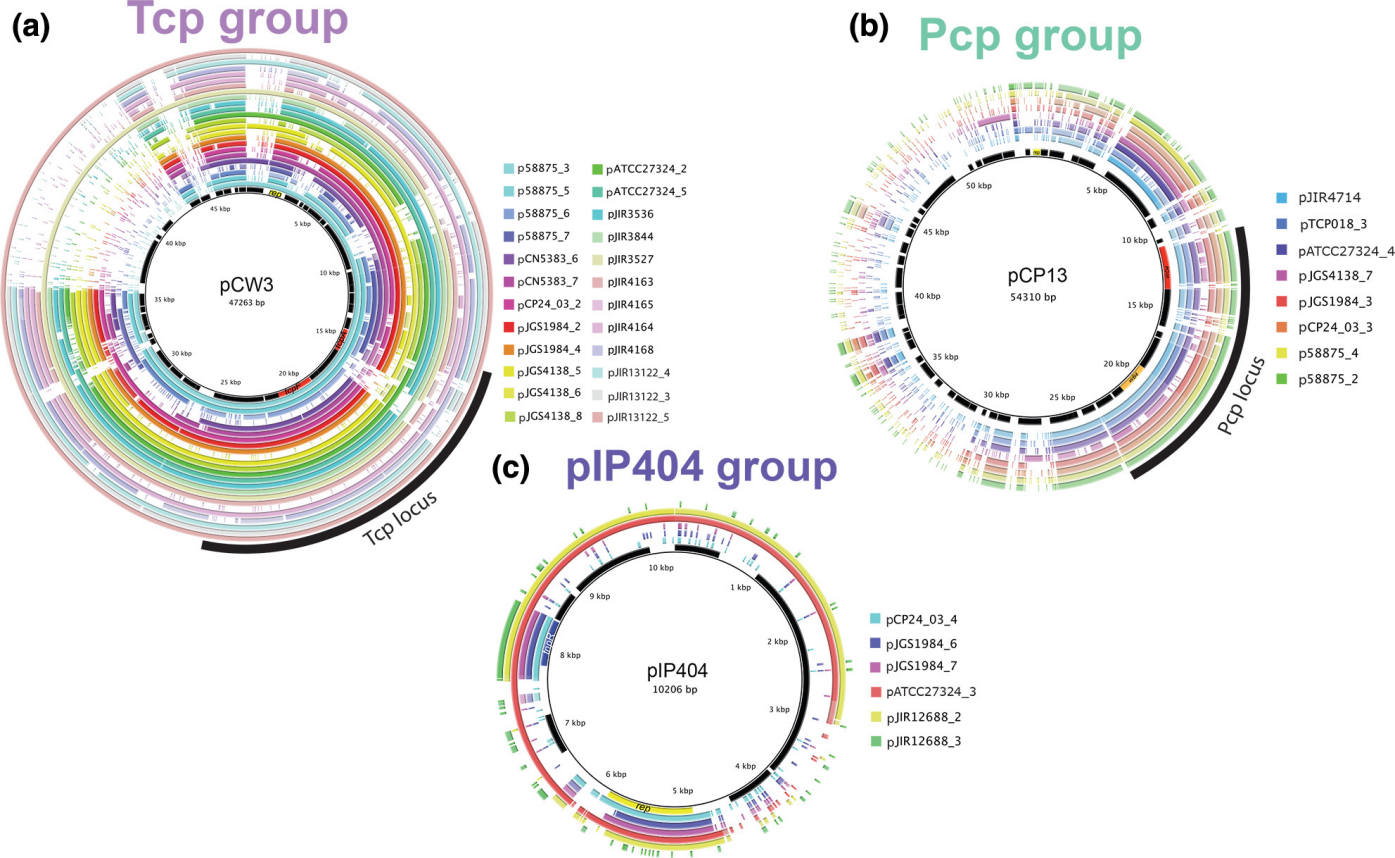
Alignments of sequenced plasmid to the three known plasmid types, Tcp, Pcp and pIP404. BRIG diagrams depicting the 24 Tcp plasmids (outer coloured rings) that have similarity to the well-studied pCW3 plasmid (inner ring annotation), eight Pcp plasmids (outer coloured rings) which shared identity with pCP13 (inner ring annotation) and six pIP404 plasmids (outer coloured rings) that share sequence with pIP404 (inner ring annotation).

**Fig. 4.**
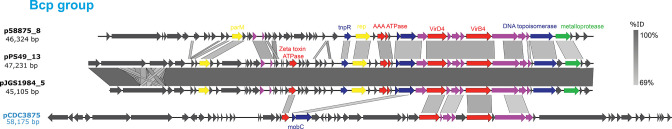
Alignment of Bcp group plasmids. An alignment of the Bcp group of plasmids p58875_8 and pJGS1984_5 and the plasmid-encoding contig pPS49_13 that contain *virD4, virB4* and *mobC* genes that are similar to those in the *C. botulinum* pCDC3875 (blue text).

**Fig. 5.**
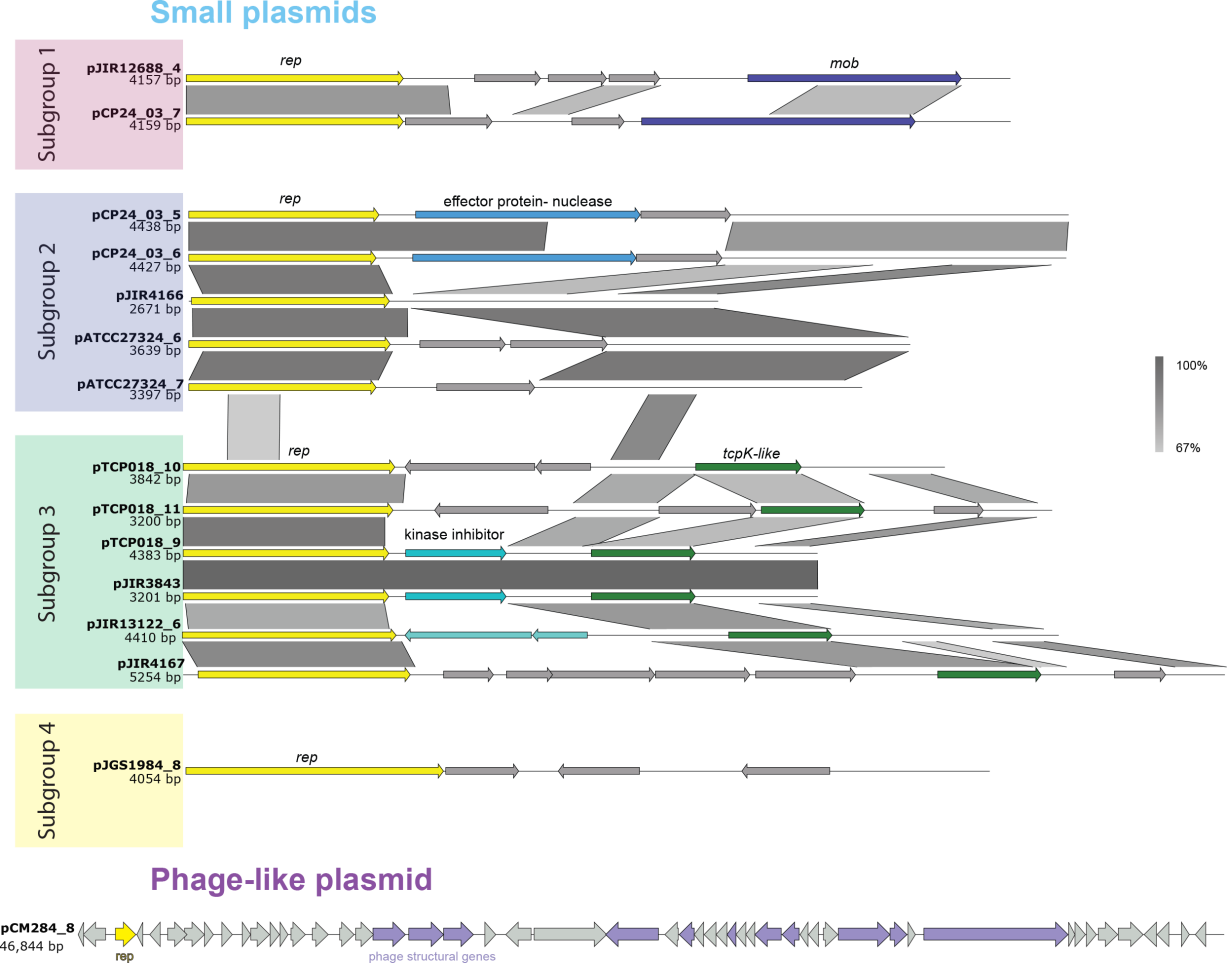
Alignment of small plasmids and genetic arrangement of the phage-like plasmid. An alignment of the small plasmids and the phage-like plasmid. Each plasmid contains a *rep* gene (yellow arrow). Four subgroups of small plasmids formed based on the similarity of their *rep* genes: subgroup 1 (red box), subgroup 2 (blue box), subgroup 3 (green box) and subgroup 4 (yellow box).

To understand the plasmid diversity present within these representative strains, the plasmids were classified into six distinct groups: Tcp plasmids (24 plasmids, 43.6 %), which contained homologues of the conjugation locus found in pCW3 (Fig. 3a); Pcp plasmids (eight plasmids, 14.5 %), which contained a conjugation locus like that from pCP13 (Fig. 3b); pIP404 plasmids (six plasmids, 10.9 %), which showed similarity to the small bacteriocin plasmid, pIP404 (Fig. 3c); the novel Bcp group (*botulinum* conjugation in *perfringens*) (two plasmids, 3.6 %), which encoded a novel conjugation locus similar to that seen in *Clostridium botulinum* (Fig. 4); a phage-like group (one plasmid, 1.8 %) (Fig. 5); and the small plasmid group (14 plasmids, 25.4 %) (Fig. 5).

The latter small plasmid group ranged in size from 2671 to 5254 bp and could be further broken down into four sub-groups, based on the similarity of their *rep* genes (Fig. 5). The first sub-group was found to carry *spoIIIE* or *ftsK* genes, respectively. SpoIIIE and FtsK are dsDNA transporters [62]: SpoIIIE is well characterized in *Bacillus subtilis* where it transports DNA through the septum and into the forespore during sporulation [63], whereas FtsK has been characterized in *Escherichia coli* where it transports DNA into a daughter cell during cell replication [63]. The FtsK/SpoIIIE homologue, TcpA*,* is commonly encoded by Tcp plasmids as a part of the Tcp conjugation locus [64]. TcpA is believed to represent the functional equivalent of the VirD4 T4SS coupling protein [65], an essential DNA transporter allowing for conjugation to occur. The presence of *ftsk*/*spoIIIE*-related genes within the first subgroup of small plasmids suggests that these plasmids may be mobilizable [65]. The second sub-group of small plasmids (pCP24_03_5, pCP24_03_6, pATCC27324_6 and pATCC27324_7) shared a large non-coding region. This non-coding region was examined for the presence of pseudogenes, small RNA sequences or an *oriT* site, but none were identified.

All plasmids in the third sub-group (pTCP018_9, pTCP018_10, pTCP018_11, pJIR13122_6 and pJIR3843) came from strains that were isolated from birds and shared a similar hypothetical gene. Through protein structure analysis (HHpred [66]) this hypothetical gene was predicted to encode a TcpK-like protein (E-value=2×10^−27^). In Tcp plasmids, TcpK is a DNA binding protein that binds to the *oriT* site, is encoded within the Tcp conjugation locus and is required for conjugation [67]. A Tcp-like *oriT* site was not identified in any of these plasmids, but *oriT* sites are notoriously difficult to identify and this finding does not preclude these plasmids from being mobilizable [15]. The final small plasmid, pJGS1984_8, contained a distinct *rep* gene from the other small plasmids. The plasmid contained a relaxase gene, which have been shown to aid in plasmid mobilization, but this gene lacks similarity to any relaxase genes found on other *C. perfringens* plasmids.

**Fig. 6.**
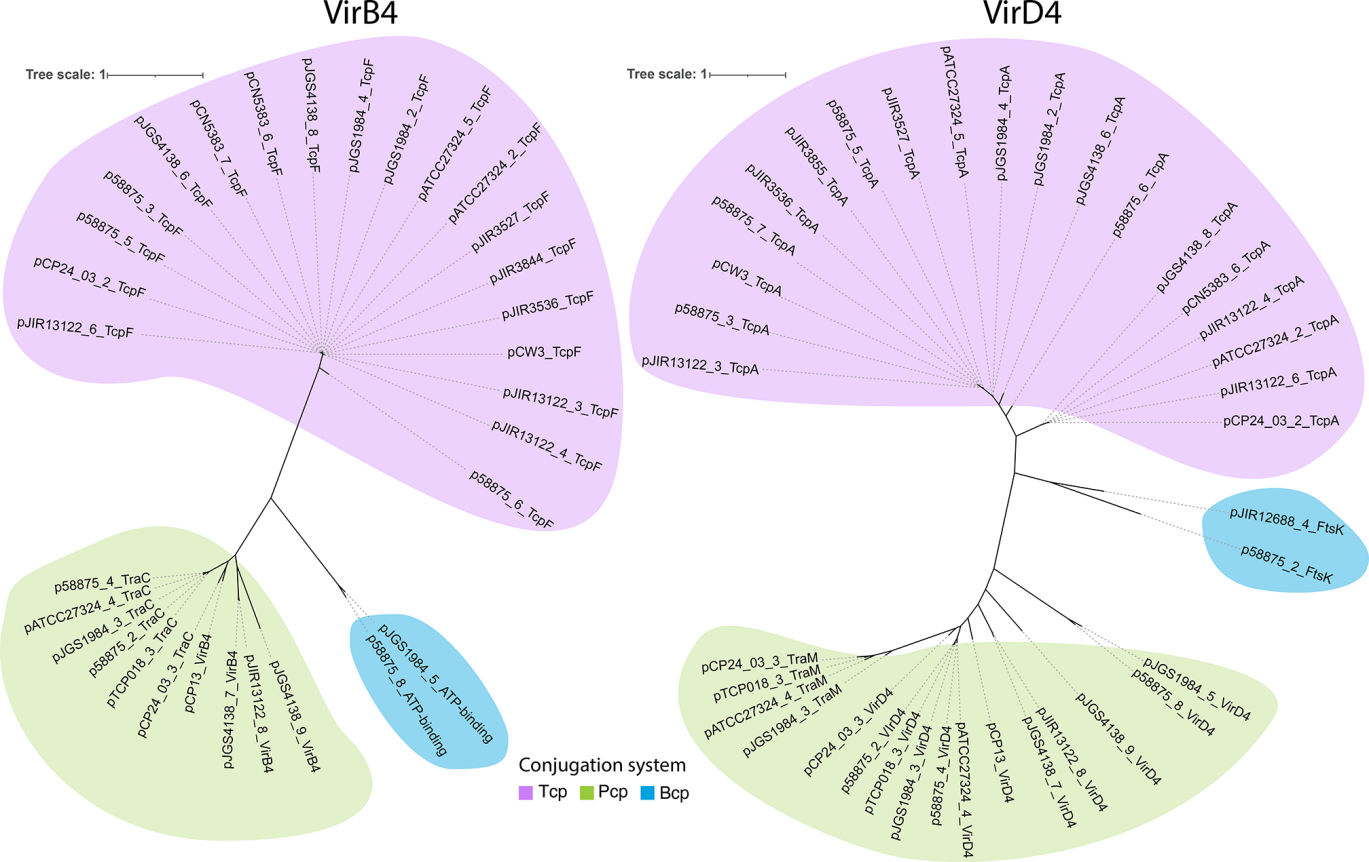
Identification of a third conjugation locus, Bcp. Maximum-likelihood trees of the (**a**) VirB4 and (**b**) VirD4 conjugation proteins identified as being encoded by the closed plasmids, where scale is branch length which is defined as base pair substitutions per sequence site. Each was categorized as being from the Tcp conjugation group (purple), the Pcp conjugation group (green) or the novel Bcp conjugation group (blue), where coloured bubbles represent sequences with at least 40 % sequence identity for VirB4 and 20 % for VirD4.

The novel Bcp group plasmids p58875_8 and pJGS1984_5 (Fig. 4) encoded proteins homologous to VirB4 and VirD4-like conjugation proteins that were distinct from the Tcp- and Pcp-encoded variants (<30 % amino acid sequence identity, Fig. 6). However, the novel *virB4* and *virD4* genes exhibited similarity to homologues from the *C. botulinum* plasmid pCDC3875 (Fig. 4). The *virD4* genes from p58875_8 and pJGS1984_5 had 57.1 and 55.4% identity, respectively, to the *virD4* gene from pCDC3875. In addition, the *virB4* genes from p58875_8 and pJGS1984_5 had 68.12 and 70.38% identity to their pCDC3875 homologues, respectively. The *C. botulinum* plasmid pCDC3875 has not been shown experimentally to be conjugative, though it contains genes sufficient for conjugation to occur [68]. Therefore, similar to Tcp and Pcp systems, it is possible that the Bcp plasmids may also be conjugative.

### Novel *C. perfringens* plasmids can be found in the large whole genome collection

**Fig. 7.**
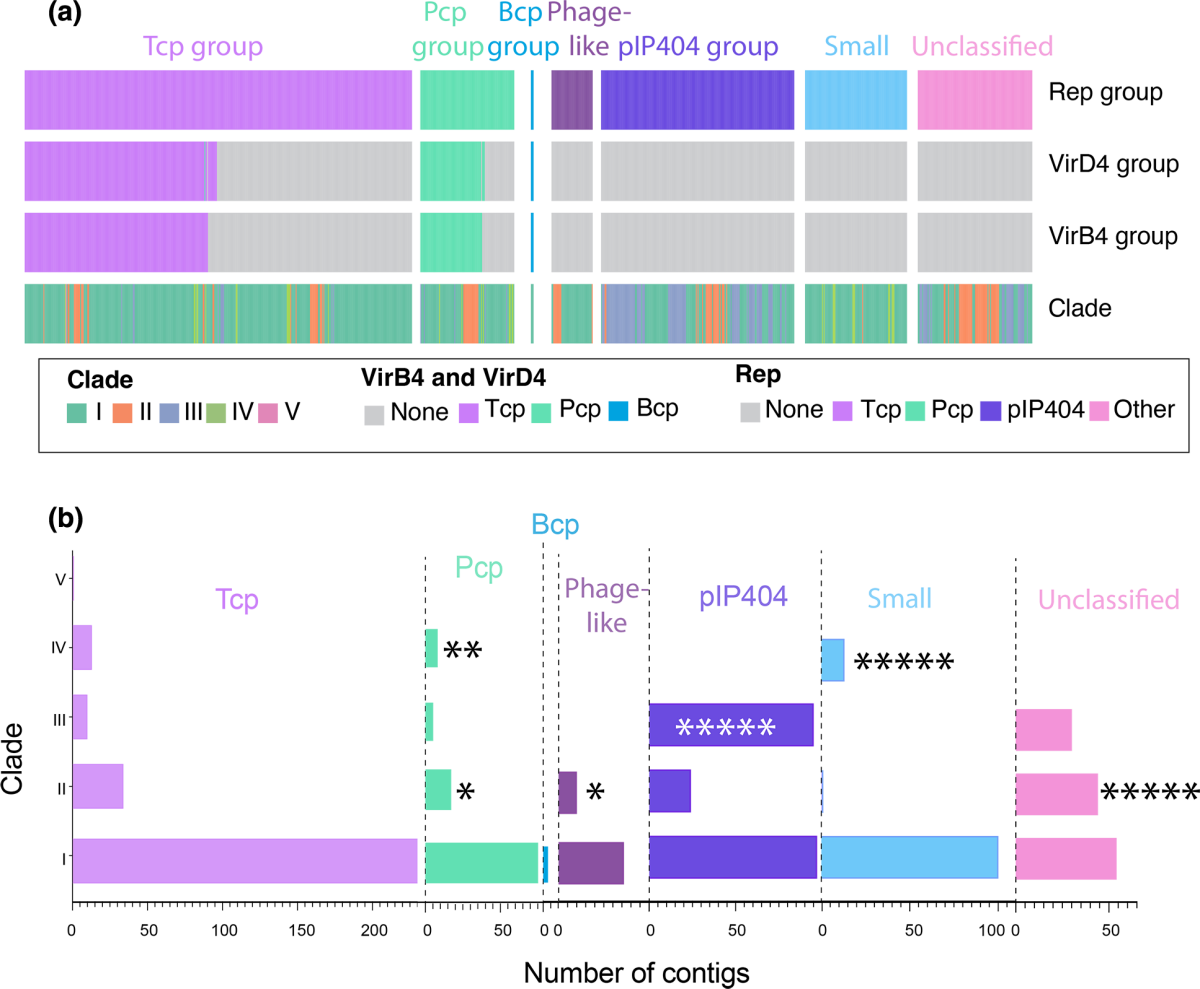
Categorization of plasmid-encoding contigs of *C. perfringens.* (a) Similarity to known VirD4, VirB4 and Rep proteins allowed for categorization of plasmid-contigs into seven groups: Tcp, Pcp, Bcp, Phage-like, pIP404, small and unclassified. (**b**) The number of each type of plasmid contig per clade. Overrepresented groups, Fisher’s exact test: *P<0.05, **P<0.01, *****P<0.00001.

To examine the prevalence of plasmids within the larger dataset, 1045 contigs that encoded either a plasmid replication (*rep*) gene or plasmid-encoded toxin genes were extracted from the 464 genomes. These contigs were able to be categorized into seven families: Tcp, Pcp, Bcp, pIP404, phage-like, small plasmids or an unclassified group (Fig. 7a). All of these plasmid-encoding contigs henceforth will be referred to as contigs, since without closed genomes it was not known if each contig represented a whole plasmid. The most common contig type was Tcp (433 contigs, 41.4 %), followed by pIP404 (216 contigs, 20.7 %), unclassified (128 contigs, 12.3 %), small plasmid (114 contigs, 10.9 %), Pcp (105 contigs, 10.1 %), phage-like (46 contigs, 4.4 %) and Bcp (three contigs, 0.3 %) (Fig. 7a).

To further characterize the content of these plasmid contigs, they were investigated for the presence of toxin, bacteriocin and antimicrobial resistance genes (Fig. S1). Toxin genes were found in the Tcp (*netG, netE, netF, netB, cpb2, itx, etx, lamda, cpe, cpb* and *tpeL*), Pcp (*netB, cpb2* and *cpb*), pIP404 (*cpb2*), small (*netB*) and uncharacterized groups (*netG, netE, netF, netB* and *cpe*). Similarly, bacteriocin genes were identified in the Pcp, pIP404 and unclassified groups. Finally, antimicrobial resistance genes were carried on contigs within the Tcp [*tetAB*(P) and *catP*] group (Fig. S1).

Examination of all the plasmid-encoded contigs has identified one additional Bcp-encoding sequence, pPS49_13, which was very similar to pJGS1984_5, with only one gene different between the two plasmids (Fig. 4). The contig also has the same plasmid mobilization, *mobC,* gene and zeta toxin-encoding gene as pJGS1984_5. Therefore, three examples of the Bcp-type putative conjugation locus within the *C. perfringens* isolates have been identified from the current study.

We next sought to understand the distribution of plasmid-like contigs by examining the location of their parent strains within the *C. perfringens* phylogeny (Fig. 7b). Clade I contained contigs from each of the seven plasmid groups, clade II contained all except Bcp, clade III contained Tcp, Pcp, pIP404 and unclassified, clade IV contained Tcp, Pcp and small contigs, and clade V contained one Tcp contig. While the contig diversity was comparable to the strain abundance within each clade, there was enrichment within clade II for Pcp contigs (17/105 Pcp contigs, Fisher’s exact test *P=*0.03), Phage-like contigs (10/46 phage-like contigs, Fisher’s exact test *P*=0.0182) and unclassified contigs (44/128 unclassified contigs, Fisher’s exact test *P*<0.00001), and an overrepresentation within clade III for pIP404 contigs (95/216 pIP404 contigs, Fisher’s exact test *P*<0.0001). Finally, clade IV had an overrepresentation of Pcp contigs (8/105 Pcp contigs, Fischer’s exact test *P*=0.005) and small contigs (13/114 small contigs, Fisher’s exact test *P*<0.00001). Therefore, intraclade plasmid-encoding contig diversity reflects the number of strains found within each clade. However, there is enrichment within clades II, III and IV for specific plasmid types, which may contribute to diversity within *C. perfringens* strains.

## Discussion

This study identified a novel conjugation locus within *C. perfringens* plasmids through the addition of a diverse group of 102 new isolates to the *C. perfringens* genome collection, including the first closed genome sequences for isolates from toxinotypes B and D. It has also added the first sequences of strains isolated from llamas and has almost doubled the collection of strains isolated from turkeys [21]. Phylogenetic relationships were maintained when comparing whole genomes as opposed to core genomes, despite the accessory genome representing half of the genes within each strain. While phylogenetic clades are the best indication of genetic relatedness between strains, they only partially align with source or disease phenotypes, which are important considerations during a disease outbreak. Through our whole genome analysis, we observed that plasmid and chromosomally encoded toxin genes correlate with the host or source of the isolate, and are necessary for the disease type. This finding is concordant with other studies showing that toxins can be the sole cause of the disease type, such as the introduction of the *netB* toxin into commensal strains, which can allow for disease pathogenesis in chickens [69]. Together, these data provide evidence that, where the nature of the causative organism is important, toxinotyping should remain as the method used to group strains, rather than phylogeny; however, phylogenetic information is still required for strain tracking through an outbreak.

Since previous genomic analyses of *C. perfringens* [10, 26, 27, 70] largely ignored plasmid sequences, we performed hybrid long- and short-read sequencing to assemble and close 55 plasmid sequences. A group of plasmids, the Bcp plasmids, were identified which encode a putative novel conjugation locus. These plasmids encoded homologues of the Type IV Secretion System (T4SS) VirD4 coupling and DNA binding protein, and the VirB4 ATPase, with <30 % identity to the corresponding proteins encoded within the Tcp or Pcp conjugation systems. However, these homologues showed identity to *C. botulinum* proteins on the botulinum neurotoxin-encoding pCDC3875 (Fig. 4) [68]. In addition to having similarity to these *virB4* and *virD4* genes, two of these plasmids had a similar zeta toxin ATPase gene to that on pCDC3875 and all of these plasmids shared a related *mobC* gene and five hypothetical genes, which flanked the *virB4* and *virD4* genes. These data indicate that the Bcp group of plasmids were derived from a common clostridial toxin plasmid lineage, which is distinct from the Tcp lineage, which is widely disseminated only amongst *C. perfringens* strains [14]. Previous work has indicated that botulinum neurotoxin-carrying *C. botulinum* plasmids can be conjugatively transferred to other clostridial members, including *Clostridium sporogenes* and *Clostridium butyricum* [71]. Therefore, there is a potential mechanism by which the *C. botulinum* conjugation system can be introduced into *C. perfringens* and recombine with existing plasmids. Although further work is required to determine if this locus encodes a functional conjugation locus, we have identified a novel putative conjugation system, only the third such locus identified in *C. perfringens.*

The collection of 55 closed plasmids included two highly similar (99.74 % nucleotide identity), non-conjugative *cpe* plasmids, pJGS4138_5 and pJIR4165. These plasmids lacked full conjugation loci, though they did encode the relaxase gene, *tcpM,* and the pCW3 *oriT,* making them likely to be mobilizable by co-resident Tcp plasmids. Previous studies have only identified *cpe* genes on the chromosome or on large conjugative plasmids [72]; consequently, these two plasmids are the first example of potentially non-conjugative *cpe-*encoding plasmids. In addition, our data show that members of three of the four small plasmids groups encode some mobilization genes, and although *oriT* sites were not identified, it remains plausible that some of these small plasmids could be mobilized, particularly as they reside within cells harbouring multiple, large conjugative plasmids [73, 74].

Our analysis also identified 46 phage-like plasmid contigs with similarity to the closed plasmid pCN5383_8, which encodes a plasmid replication gene as well as many phage structural genes (Fig. 5). Genetic elements carrying both plasmid and phage genes have been previously identified in other species and are known as phage-plasmids [75]. Despite *C. perfringens* plasmids and phages being well studied, and phage-plasmids being very common in other clostridia, this is one of the few characterized phage-plasmids within *C. perfringens.*

This study has identified plasmids which carry key toxin genes that are known to be the causative agent of specific disease pathologies. Through the characterization of these plasmids, we have identified a novel, putative, conjugative apparatus that may be contributing to the dissemination of genes within the species and between *C. perfringens* and other species, specifically *C. botulinum.* Furthermore, this study has emphasized the lack of understanding of many plasmid-encoded genes. The 55 plasmids studied in detail encoded 2852 genes, of which at least 1162 (40.7 %) were hypothetical or had an unknown function. Given the average number of plasmids found within each of the 464 strains examined was two, with a maximum of 10 identified, there is a wealth of hypothetical genes that are yet to be examined. Consequently, although plasmid research has enabled characterization of conjugation, toxin and antimicrobial resistance genes, there is still a lot of work to be done in characterizing other plasmid-encoded genes to fully understand how plasmids within *C. perfringens* move, replicate and regulate functions important to host cell pathogenesis.

The genome collection of *C. perfringens* has been expanded from 362 to 464 with a diverse group of isolates from a broad host range, and we have utilized this collection to understand the phenotypic diversity exemplified by the species. It was shown that accessory genes, particularly those encoded by plasmids, are the main drivers of disease phenotypes. Furthermore, the detailed characterization of plasmid types allowed for the identification of the novel Bcp conjugation locus for the first time.

## Supplementary Data

Supplementary material 1

Supplementary material 2

## References

[1] Sarker MR, Carman RJ, McClane BA (1999). Inactivation of the gene (*cpe*) encoding *Clostridium perfringens* enterotoxin eliminates the ability of two *cpe*-positive *C. perfringens* type A human gastrointestinal disease isolates to affect rabbit ileal loops. Mol Microbiol.

[2] Uzal FA, Freedman JC, Shrestha A, Theoret JR, Garcia J (2014). Towards an understanding of the role of *Clostridium perfringens* toxins in human and animal disease. Future Microbiol.

[3] Awad MM, Bryant AE, Stevens DL, Rood JI (1995). Virulence studies on chromosomal alpha-toxin and theta-toxin mutants constructed by allelic exchange provide genetic evidence for the essential role of alpha-toxin in *Clostridium perfringens*-mediated gas gangrene. Mol Microbiol.

[4] Blackwell TE, Butler DG, Prescott JF, Wilcock BP (1991). Differences in signs and lesions in sheep and goats with enterotoxemia induced by intraduodenal infusion of *Clostridium perfringens* type D. Am J Vet Res.

[5] Lee KW, Lillehoj HS (2021). Role of *Clostridium perfringens* necrotic enteritis B-like toxin in disease pathogenesis. Vaccines.

[6] Mehdizadeh Gohari I, Parreira VR, Nowell VJ, Nicholson VM, Oliphant K (2015). A novel pore-forming toxin in type A *Clostridium perfringens* is associated with both fatal canine hemorrhagic gastroenteritis and fatal foal necrotizing enterocolitis. PLoS One.

[7] Shao Y, Forster SC, Tsaliki E, Vervier K, Strang A (2019). Stunted microbiota and opportunistic pathogen colonization in caesarean-section birth. Nature.

[8] Carman RJ, Sayeed S, Li J, Genheimer CW, Hiltonsmith MF (2008). *Clostridium perfringens* toxin genotypes in the feces of healthy North Americans. Anaerobe.

[9] Rood JI, Adams V, Lacey J, Lyras D, McClane BA (2018). Expansion of the *Clostridium perfringens* toxin-based typing scheme. Anaerobe.

[10] Geier RR, Rehberger TG, Smith AH (2021). Comparative genomics of *Clostridium perfringens* reveals patterns of host-associated phylogenetic clades and virulence factors. Front Microbiol.

[11] Uzal FA (2004). Diagnosis of *Clostridium perfringens* intestinal infections in sheep and goats. Anaerobe.

[12] Watts TD, Johanesen PA, Lyras D, Rood JI, Adams V (2017). Evidence that compatibility of closely related replicons in *Clostridium perfringens* depends on linkage to parMRC-like partitioning systems of different subfamilies. Plasmid.

[13] Garnier T, Cole ST (1986). Characterization of a bacteriocinogenic plasmid from *Clostridium perfringens* and molecular genetic analysis of the bacteriocin-encoding gene. J Bacteriol.

[14] Li J, Adams V, Bannam TL, Miyamoto K, Garcia JP (2013). Toxin plasmids of *Clostridium perfringens*. Microbiol Mol Biol Rev.

[15] Bannam TL, Teng WL, Bulach D, Lyras D, Rood JI (2006). Functional identification of conjugation and replication regions of the tetracycline resistance plasmid pCW3 from *Clostridium perfringens*. J Bacteriol.

[16] Mehdizadeh Gohari I, A Navarro M, Li J, Shrestha A, Uzal F (2021). Pathogenicity and virulence of *Clostridium perfringens*. Virulence.

[17] Adams V, Han X, Lyras D, Rood JI (2018). Antibiotic resistance plasmids and mobile genetic elements of *Clostridium perfringens*. Plasmid.

[18] Revitt-Mills SA, Vidor CJ, Watts TD, Lyras D, Rood JI (2019). Virulence plasmids of the pathogenic *Clostridia*. Microbiol Spectr.

[19] Watts TD, Vidor CJ, Awad MM, Lyras D, Rood JI (2019). pCP13, a representative of a new family of conjugative toxin plasmids in *Clostridium perfringens*. Plasmid.

[20] Yonogi S, Matsuda S, Kawai T, Yoda T, Harada T (2014). BEC, a novel enterotoxin of *Clostridium perfringens* found in human clinical isolates from acute gastroenteritis outbreaks. Infect Immun.

[21] Lacey JA, Allnutt TR, Vezina B, Van TTH, Stent T (2018). Whole genome analysis reveals the diversity and evolutionary relationships between necrotic enteritis-causing strains of *Clostridium perfringens*. BMC Genomics.

[22] Carey J, Cole J, Venkata SLG, Hoyt H, Mingle L (2021). Determination of genomic epidemiology of historical *Clostridium perfringens* outbreaks in New York state by use of two web-based platforms: national center for biotechnology information pathogen detection and FDA galaxytrakr. J Clin Microbiol.

[23] Fourie JCJ, Bezuidenhout CC, Sanko TJ, Mienie C, Adeleke R (2020). Inside environmental *Clostridium perfringens* genomes: antibiotic resistance genes, virulence factors and genomic features. J Water Health.

[24] Kiu R, Brown J, Bedwell H, Leclaire C, Caim S (2019). Genomic analysis on broiler-associated *Clostridium perfringens* strains and exploratory caecal microbiome investigation reveals key factors linked to poultry necrotic enteritis. Anim Microbiome.

[25] Mahamat Abdelrahim A, Radomski N, Delannoy S, Djellal S, Le Négrate M (2019). Large-scale genomic analyses and toxinotyping of *Clostridium perfringens* implicated in foodborne outbreaks in France. Front Microbiol.

[26] Abdel-Glil MY, Thomas P, Linde J, Busch A, Wieler LH (2021). Comparative *in silico* genome analysis of *Clostridium perfringens* unravels stable phylogroups with different genome characteristics and pathogenic potential. Sci Rep.

[27] Kiu R, Caim S, Alexander S, Pachori P, Hall LJ (2017). Probing genomic aspects of the multi-host pathogen *Clostridium perfringens* reveals significant pangenome diversity, and a diverse array of virulence factors. Front Microbiol.

[28] Camargo A, Guerrero-Araya E, Castañeda S, Vega L, Cardenas-Alvarez MX (2022). Intra-species diversity of *Clostridium perfringens*: a diverse genetic repertoire reveals its pathogenic potential. Front Microbiol.

[29] Browne HP, Forster SC, Anonye BO, Kumar N, Neville BA (2016). Culturing of “unculturable” human microbiota reveals novel taxa and extensive sporulation. Nature.

[30] Abraham LJ, Rood JI (1985). Molecular analysis of transferable tetracycline resistance plasmids from *Clostridium perfringens*. J Bacteriol.

[31] O’Connor JR, Lyras D, Farrow KA, Adams V, Powell DR (2006). Construction and analysis of chromosomal *Clostridium difficile* mutants. Mol Microbiol.

[32] Wick RR, Judd LM, Gorrie CL, Holt KE (2017). Unicycler: resolving bacterial genome assemblies from short and long sequencing reads. PLoS Comput Biol.

[33] Parks DH, Imelfort M, Skennerton CT, Hugenholtz P, Tyson GW (2015). CheckM: assessing the quality of microbial genomes recovered from isolates, single cells, and metagenomes. Genome Res.

[34] Challis R (2017). Rjchallis/assembly-stats. Zenodo.

[35] Chaumeil P-A, Mussig AJ, Hugenholtz P, Parks DH (2022). GTDB-Tk v2: memory friendly classification with the genome taxonomy database. Bioinformatics.

[36] Schwengers O, Jelonek L, Dieckmann MA, Beyvers S, Blom J (2021). Bakta: rapid and standardized annotation of bacterial genomes via alignment-free sequence identification. Microb Genom.

[37] Karp PD, Billington R, Caspi R, Fulcher CA, Latendresse M (2019). The BioCyc collection of microbial genomes and metabolic pathways. Brief Bioinform.

[38] Page AJ, Cummins CA, Hunt M, Wong VK, Reuter S (2015). Roary: rapid large-scale prokaryote pan genome analysis. Bioinformatics.

[39] Stamatakis A (2014). RAxML version 8: a tool for phylogenetic analysis and post-analysis of large phylogenies. Bioinformatics.

[40] Katz LS, Griswold T, Morrison SS, Caravas JA, Zhang S (2019). Mashtree: a rapid comparison of whole genome sequence files. J Open Source Softw.

[41] Letunic I, Bork P (2021). Interactive Tree Of Life (iTOL) v5: an online tool for phylogenetic tree display and annotation. Nucleic Acids Res.

[42] Buchfink B, Reuter K, Drost HG (2021). Sensitive protein alignments at tree-of-life scale using DIAMOND. Nat Methods.

[43] Seeman T Abricate. https://githubcom/tseemann/abricate..

[44] Zankari E, Hasman H, Cosentino S, Vestergaard M, Rasmussen S (2012). Identification of acquired antimicrobial resistance genes. J Antimicrob Chemother.

[45] Sullivan MJ, Petty NK, Beatson SA (2011). Easyfig: a genome comparison visualizer. Bioinformatics.

[46] Alikhan N-F, Petty NK, Ben Zakour NL, Beatson SA (2011). BLAST Ring Image Generator (BRIG): simple prokaryote genome comparisons. BMC Genomics.

[47] Katoh K, Standley DM (2013). MAFFT multiple sequence alignment software version 7: improvements in performance and usability. Mol Biol Evol.

[48] Price MN, Dehal PS, Arkin AP (2010). FastTree 2--approximately maximum-likelihood trees for large alignments. PLoS One.

[49] Altschul SF, Gish W, Miller W, Myers EW, Lipman DJ (1990). Basic local alignment search tool. J Mol Biol.

[50] Orrell KE, Melnyk RA (2021). Large clostridial toxins: mechanisms and roles in disease. Microbiol Mol Biol Rev.

[51] Fernandez-Miyakawa ME, Fisher DJ, Poon R, Sayeed S, Adams V (2007). Both epsilon-toxin and beta-toxin are important for the lethal properties of *Clostridium perfringens* type B isolates in the mouse intravenous injection model. Infect Immun.

[52] Uzal FA, Songer JG (2008). Diagnosis of *Clostridium perfringens* intestinal infections in sheep and goats. J Vet Diagn Invest.

[53] Uzal FA, Kelly WR (1998). Experimental *Clostridium perfringens* type D enterotoxemia in goats. Vet Pathol.

[54] Scallan E, Hoekstra RM, Angulo FJ, Tauxe RV, Widdowson M-A (2011). Foodborne illness acquired in the United States--major pathogens. Emerg Infect Dis.

[55] Mehdizadeh Gohari I, Kropinski AM, Weese SJ, Whitehead AE, Parreira VR (2017). NetF-producing *Clostridium perfringens*: clonality and plasmid pathogenicity loci analysis. Infect Genet Evol.

[56] Revitt-Mills SA, Rood JI, Adams V (2015). *Clostridium perfringens* extracellular toxins and enzymes: 20 and counting. Microbiol Aust.

[57] Han X, Du X-D, Southey L, Bulach DM, Seemann T (2015). Functional analysis of a bacitracin resistance determinant located on ICECp1, a novel Tn916-like element from a conjugative plasmid in *Clostridium perfringens*. Antimicrob Agents Chemother.

[58] Bannam TL, Yan X-X, Harrison PF, Seemann T, Keyburn AL (2011). Necrotic enteritis-derived *Clostridium perfringens* strain with three closely related independently conjugative toxin and antibiotic resistance plasmids. mBio.

[59] Miyamoto K, Fisher DJ, Li J, Sayeed S, Akimoto S (2006). Complete sequencing and diversity analysis of the enterotoxin-encoding plasmids in *Clostridium perfringens* type A non-food-borne human gastrointestinal disease isolates. J Bacteriol.

[60] Manich M, Knapp O, Gibert M, Maier E, Jolivet-Reynaud C (2008). *Clostridium perfringens* delta toxin is sequence related to beta toxin, NetB, and Staphylococcus pore-forming toxins, but shows functional differences. PLoS One.

[61] Li J, Miyamoto K, Sayeed S, McClane BA (2010). Organization of the CPE locus in CPE-positive *Clostridium perfringens* type C and D isolates. PLoS One.

[62] Barre FX (2007). FtsK and SpoIIIE: the tale of the conserved tails. Mol Microbiol.

[63] Wu LJ, Errington J (1994). *Bacillus subtilis* SpoIIIE protein required for DNA segregation during asymmetric cell division. Science.

[64] Parsons JA, Bannam TL, Devenish RJ, Rood JI (2007). TcpA, an FtsK/SpoIIIE homolog, is essential for transfer of the conjugative plasmid pCW3 in *Clostridium perfringens*. J Bacteriol.

[65] Parsons JA, Bannam TL, Devenish RJ, Rood JI (2007). TcpA, an FtsK/SpoIIIE homolog, is essential for transfer of the conjugative plasmid pCW3 in *Clostridium perfringens*. J Bacteriol.

[66] Zimmermann L, Stephens A, Nam S-Z, Rau D, Kübler J (2018). A completely reimplemented MPI bioinformatics toolkit with a new HHpred server at its core. J Mol Biol.

[67] Traore DAK, Wisniewski JA, Flanigan SF, Conroy PJ, Panjikar S (2018). Crystal structure of TcpK in complex with oriT DNA of the antibiotic resistance plasmid pCW3. Nat Commun.

[68] Carter AT, Austin JW, Weedmark KA, Corbett C, Peck MW (2014). Three classes of plasmid (47-63 kb) carry the type B neurotoxin gene cluster of group II *Clostridium botulinum*. Genome Biol Evol.

[69] Lacey JA, Keyburn AL, Ford ME, Portela RW, Johanesen PA (2017). Conjugation-mediated horizontal gene transfer of *Clostridium perfringens* plasmids in the chicken gastrointestinal tract results in the formation of new virulent strains. Appl Environ Microbiol.

[70] Feng Y, Fan X, Zhu L, Yang X, Liu Y (2020). Phylogenetic and genomic analysis reveals high genomic openness and genetic diversity of *Clostridium perfringens*. Microb Genom.

[71] Nawrocki EM, Bradshaw M, Johnson EA (2018). Botulinum neurotoxin-encoding plasmids can be conjugatively transferred to diverse clostridial strains. Sci Rep.

[72] Brynestad S, Sarker MR, McClane BA, Granum PE, Rood JI (2001). Enterotoxin plasmid from *Clostridium perfringens* is conjugative. Infect Immun.

[73] Yui Eto K, Kwong SM, LaBreck PT, Crow JE, Traore DAK (2021). Evolving origin-of-transfer sequences on staphylococcal conjugative and mobilizable plasmids-who’s mimicking whom?. Nucleic Acids Res.

[74] Firth N, Jensen SO, Kwong SM, Skurray RA, Ramsay JP (2018). Staphylococcal plasmids, transposable and integrative elements. Microbiol Spectr.

[75] Pfeifer E, Moura de Sousa JA, Touchon M, Rocha EPC (2021). Bacteria have numerous distinctive groups of phage-plasmids with conserved phage and variable plasmid gene repertoires. Nucleic Acids Res.

