# Supplementary figures and images for: Extensive genome analysis identifies novel plasmid families in Clostridium perfringens
Source: Microb Genom. 2023 Apr 20;9(4):mgen000995. doi: 10.1099/mgen.0.000995 (PMC10210947; doi:10.1099/mgen.0.000995)

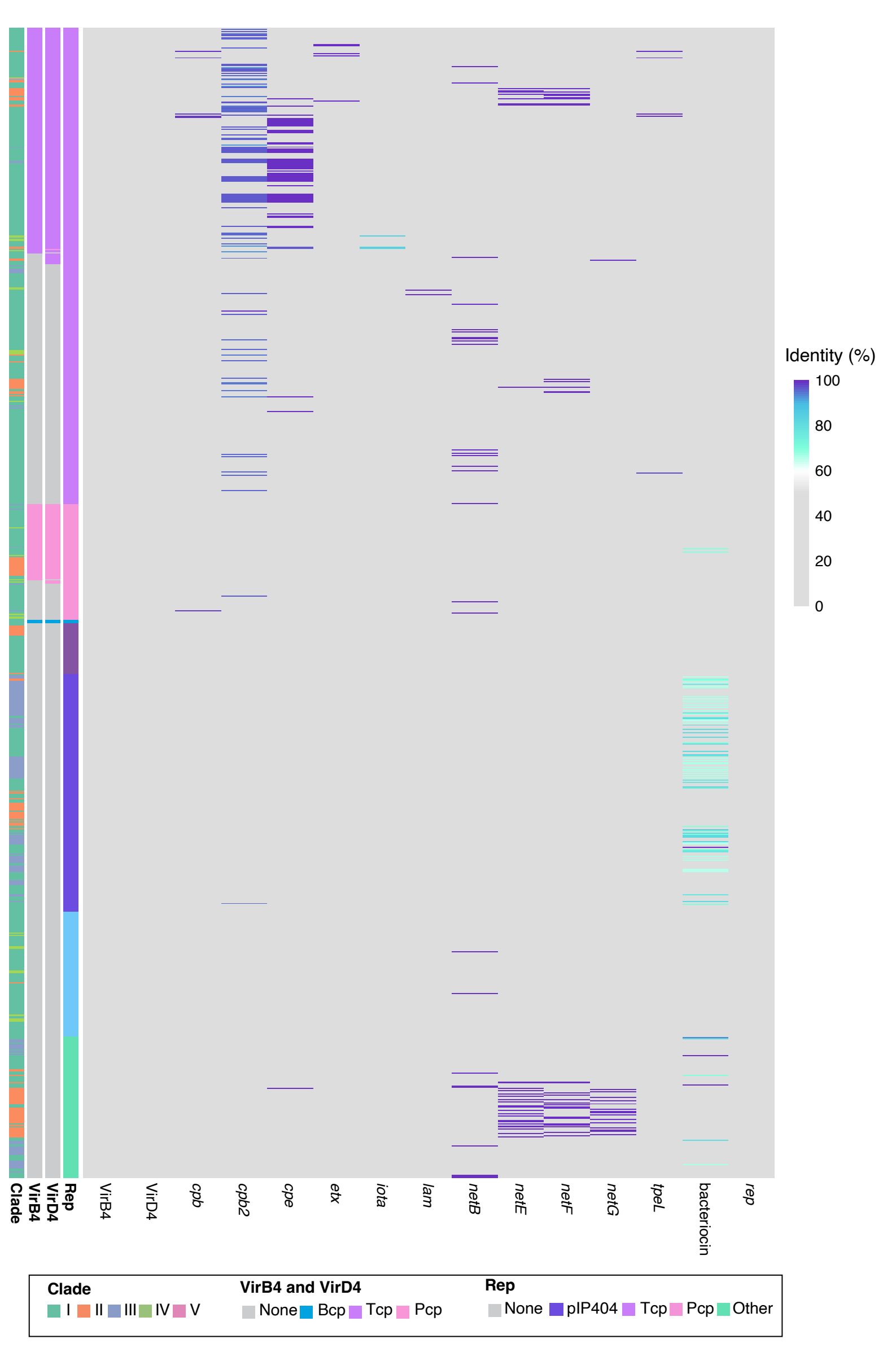

Supplement: Supplementary material 1 [file mgen-9-995-s001.pdf]
